# Effects of waterlogging on carbon assimilate partitioning in the Zoigê alpine wetlands revealed by ^13^CO_2_ pulse labeling

**DOI:** 10.1038/srep09411

**Published:** 2015-03-23

**Authors:** Jun-Qin Gao, Ju-Juan Gao, Xue-Wen Zhang, Xing-Liang Xu, Zhao-Heng Deng, Fei-Hai Yu

**Affiliations:** 1 School of Nature Conservation, Beijing Forestry University, Beijing 100083, China; 2 Key Laboratory of Ecosystem Network Observation and Modelling, Institute of Geographic Sciences and Natural Resources Research, Chinese Academy of Sciences, Beijing 100101, China

## Abstract

Waterlogging has been suggested to affect carbon (C) turnover in wetlands, but how it affects C allocation and stocks remains unclear in alpine wetlands. Using *in situ* ^13^CO_2_ pulse labelling, we investigated C allocation in both waterlogged and non-waterlogged sites in the Zoigê wetlands on the Tibetan Plateau in August 2011. More than 50% of total ^13^C fixed by photosynthesis was lost via shoot respiration. Shoots recovered about 19% of total ^13^C fixed by photosynthesis at both sites. Only about 26% of total fixed ^13^C was translocated into the belowground pools. Soil organic C pool accounted for 19% and roots recovered about 5–7% of total fixed ^13^C at both sites. Waterlogging significantly reduced soil respiration and very little ^13^C was lost via soil respiration in the alpine wetlands compared to that in grasslands. We conclude that waterlogging did not significantly alter C allocations among the C pools except the ^13^CO_2_ efflux derived from soil respiration and that shoots made similar contributions to C sequestration as the belowground parts in the Zoigê alpine wetlands. Therefore, changes in waterlogging due to climate change will not affect C assimilate partitioning but soil C efflux.

The importance of wetlands for global carbon (C) cycle and C sequestration is highlighted because a large amount of C is stored in wetland soils^1^,^2^. The amount corresponds to 20–30% of the terrestrial soil organic C (SOC, 2300 Pg) pool^1^,^2^,^3^, although wetlands only cover 5–8% of the terrestrial land surface^3^. The high C sequestration in wetland soils, compared to other ecosystems, results from the high C input by plants and very low decomposition rate of organic matter due to permanent or temporal waterlogging^3^. Waterlogging can affect plant growth and development^3^,^4^, nitrogen (N) and C mineralization^5^,^6^, microbe activities^7^,^8^,^9^ and thus C translocation among atmosphere-plant-soil-water system^10^,^11^. Previous studies on C translocation in wetlands mostly based on indirect estimates from inventories of different C pools^6^,^12^,^13^,^14^. However, direct measurements of C pools cannot adequately describe and quantify *in situ* C partitioning^15^,^16^,^17^,^18^.

^13^C isotope labeling approach is a powerful tool to quantify the partitioning of assimilated C and trace their fate in different ecosystems. It has been intensively used in crops, forests and grasslands^18^,^22^. C partitioning into roots^15^,^19^,^20^,^21^,^22^, soil^15^,^19^,^21^,^23^,^24^ and atmosphere^15^,^19^,^25^ varies widely in different ecosystems, e.g., crops can transfer 20–30% of assimilated C to the soil, meadow plants can transfer 30–50%, but trees transfer much less^18^. However, it remains unclear how plants allocate their photosynthesized C in alpine wetlands, which hinders our understanding and predicting soil organic C dynamics in alpine wetlands.

The Tibetan Plateau contains a vast area of wetlands (roughly 5.1 × 10^3^ km^2^)^26^. The Zoigê wetlands, located in the northeastern part of the Tibetan Plateau, represent the largest peatland in China^27^, which contains about 0.5 Pg C, roughly 88% of the C stocks in the Tibetan Plateau wetlands^26^. In recent decades, there is a dramatic change in water level in the Zoigê wetlands as a result of climate warming and human activities such as overgrazing and drainage^28^, leading to wetlands degradation. Decreasing water table can affect C translocation and C sequestration capacity, which is a key linkage between the C cycles in alpine wetlands and climate change. Therefore, examining how waterlogging affects C dynamics in the Zoigê wetlands is essential for estimating regional C budgets and for precisely predicting the effect of climate change on SOC sequestration and stocks in alpine wetlands. We hypothesized that wetland plants allocate newly fixed C more to belowground pools than to aboveground pools in Tibetan alpine wetlands, and that waterlogging can increase C partitioning towards to the belowground pool. To test the hypotheses, we conducted an *in situ* ^13^C labeling experiment at both waterlogged and non-waterlogged sites in the Zoigê wetlands on the Tibetan Plateau over 28 days. We aim to clarify the partitioning of recently fixed C among pools in the plant-soil system of the wetlands and the effects of waterlogging on C partitioning in alpine wetlands.

## Results

### Effects of waterlogging on ecosystem C stocks

C stocks in shoots and roots differed significantly between the waterlogged and the control (non-waterlogged) site (Table 1). In the waterlogged site, shoots stored 2.41 Mg C ha^−1^, 0.73 times higher than in the control site (1.39 Mg C ha^−1^; Table 1). The root C stock was 1.3 times higher in the waterlogged site than in the control site (Table 1). However, soil C pool, which was much greater than shoot and root C pools, did not differ significantly between the waterlogged and the control site. Additionally, waterlogging significantly decreased soil respiration (Table 1).

### Effects of waterlogging on ^13^C partitioning within pools

**Figure 1:**
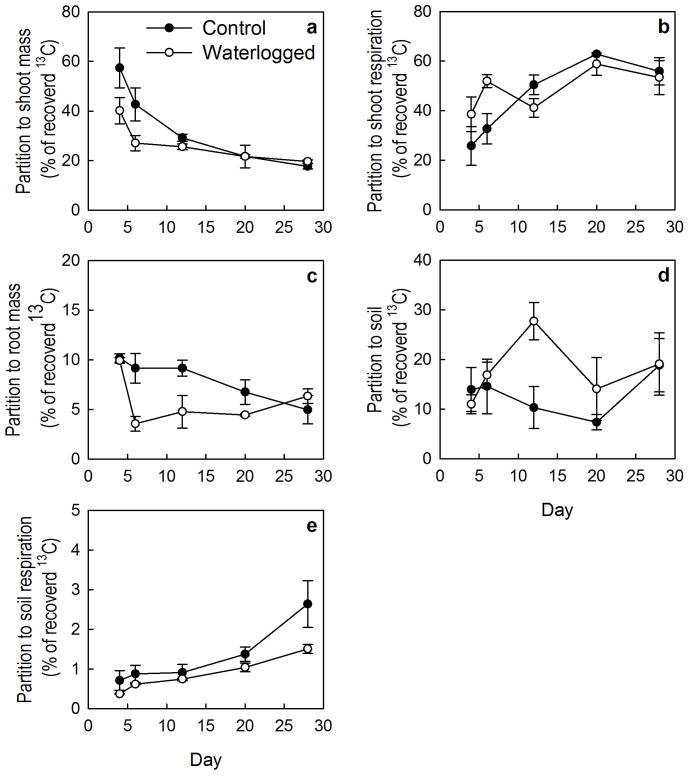
Partition of ^13^C to each of the five C pools in the control and the waterlogged site measured on days 4, 6, 12, 20, 20 and 28.

^13^C partitioning differed greatly among C pools (F = 222.0, *P* <0.001, Table 2). In the control site, ^13^C partitioning to shoot respiration (55.8 ± 5.6%) was the highest, that to soil respiration (2.6 ± 0.6%) the lowest, and that to shoot mass (17.7 ± 1.7%), soil C pool (18.8 ± 5.4%) and root mass (5.0 ± 1.4%) in between. ^13^C partitioning to shoot mass and root mass generally decreased with time (Table 2, Fig. 1a, c), but that to shoot respiration and to soil respiration increased (Table 2, Fig. 1b, e). ^13^C partitioning to soil first decreased and then increased with time (Table 2, Fig. 1d).

Effects of waterlogging on ^13^C partitioning to shoot mass, shoot respiration, root mass and soil respiration varied significantly with time (Table 3). ^13^C partitioning to shoot mass was higher in the control site than in the waterlogged site on days 4 and 6, but did not differ significantly between the two sites during the remaining period (Fig. 1a). ^13^C partitioning to shoot respiration was lower in the control site than in the waterlogged site on days 4 and 6, but such differences diminished with time (Fig. 1b). In contrast, ^13^C partitioning to soil respiration hardly differed between the waterlogged and the control site in the earlier stage, and such differences increased greatly at the end of the experiment (Fig. 1e). ^13^C partitioning to root mass was higher in the control site than in the waterlogged site on days 6, 12 and 20, but did not differ significantly on days 4 and 28 (Fig. 1c). ^13^C partitioning to soil was higher in the control site than in the waterlogged site on day 12, but did not differ significantly on other days (Fig. 1d).

## Discussion

**Figure 2:**
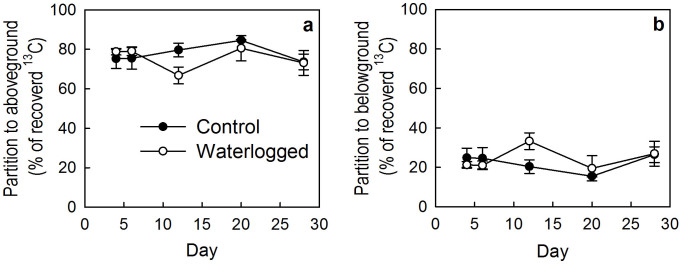
Partition of ^13^C to above- and belowground C pools in the control and the waterlogged plots measured on days 4, 6, 12, 20, 20 and 28.

The ^13^C pulse labelling approach allowed us to assess the allocation of recently fixed C to both aboveground and belowground pools under *in situ* conditions^15^,^19^. In this study we demonstrated that more than 60% of recently assimilated ^13^C was allocated to aboveground parts (Fig. 2), and about 50% was released through shoot respiration. Only less than 3% was lost via soil respiration (Fig. 1). This partitioning pattern is different from previous studies in grazed grasslands, in which most of recently assimilated ^13^C is allocated to belowground pools and more C is released back to the atmosphere via root and rhizomocrobial respiration^15^,^19^. This could be ascribed to lower soil respiration in alpine wetlands than in grasslands^29^,^30^,^31^ due to water saturation. However, our study indicates that low soil respiration in alpine wetlands could be a result of an underestimation of root respiration because a fraction of CO_2_ derived from root respiration (root and rhizomicrobial respiration) is lost via the vascular system of wetland plants^3^. This indicates that soil respiration may be major drived from old C due to high dissolved organic C in alpine wetlands, which needs to be further investigated.

Numerous studies have suggested that belowground parts played an important role for C sequestration in grasslands^15^,^23^. In this study, we showed a distinct pattern: shoot and soil pools recovered similar amounts of recent assimilated ^13^C. Higher C allocation to shoots may be ascribed to the development stage of the studied plants^32^,^33^,^34^, because most plants were flowering or even fruiting (e.g., *C. muliensis* and *C. lasiocarpa*) during the chase period. More recently assimilated ^13^C was therefore allocated to shoots for reproduction^19^. For the belowground C pools, more C was transferred to soil but less C was recovered in roots. The probable reason could be the rapid rhizosphere deposition^22^.

Waterlogging can increase biomass, decrease soil respiration but cannot significantly increase SOC storage mainly due to the continuously changeable environment, e.g., water table, and C run-off during long-term SOC accumulation period^28^. Total CO_2_ emission can be reduced by 50% due to waterlogging in the Zoigê wetlands^29^. In this study, we observed that waterlogging reduced soil respiration by 36% and soil^13^CO_2_ by 42% compared to the non-waterlogged plots (Table 1, Fig. 1). Waterlogging demonstrated complicated effects on C assimilate partitioning which relies on C pools and times. We found that shoot ^13^C decreased with time mostly because newly fixed ^13^C was transferred to the belowground part or the atmosphere. Both shoot and soil respiration increased with time because more ^13^C was consumed. Soil and root C pools changed with time irregularly, especially in the waterlogged site. This may be due to variable water table or soil moisture during the chase period. We found that soil moisture content changed in the waterlogged and control sites (Supplementary Fig. S2 online) during the chase period, which may affect soil respiration and belowground C partition. However, the speculation needs further investigations.

In conclusion, recently assimilated C can be rapidly transferred to soil via rhizosphere deposition. Compared to the belowground parts, the aboveground parts play similar role in C sequestration in alpine wetlands. Waterlogging showed a complicated effect on C assimilate partitioning which relies on C pools and time. Further studies should investigate the fate of the remaining of the recently-assimilated C to improve our understanding of the mechanisms responsible for C sequestration in alpine wetlands.

## Methods

### Site description

The field sites were in an alpine wetlands (33° 35′N, 102°57′E, 3442 m asl.) located in the eastern part of the Tibet Plateau at Zoigê County in Sichuan province, China. Annual precipitation averages 650 mm, and most occurs during the growing season from May to September^35^. Annual mean temperature is 0.6°C; the lowest mean monthly temperature is −10.7°C in January, and the highest is 10.9°C in July. Due to low temperature, litter decomposition is slow, and soil organic matter and peat accumulate gradually. The peat is in general 2–5 m depth in the Zoigê alpine wetlands^35^,^36^.

In July 2011, we established two 10 m × 10 m sites in the wetlands. One site was used as the control site where water table was about −5 cm from the soil surface. The other one was the waterlogged site where water table was about 5 cm from the soil surface, which was adjacent to the control site. The control site was dominated by *C. muliensis*, *Carex lasiocarpa* and *Caltha scaposa*, *Polygonum sibiricum*, *Potentilla anserina* and *Equisetum arvense*, and the waterlogged site was dominated by *C. muliensis*, *C. lasiocarpa* and *C. scaposa*. The field studies did not involve any endangered or protected species and no specific permits were required for the described studies.

### ^13^C pulse labeling

On 25 July 2011, in each of the two sites we set up three plots (labeled plots) for ^13^CO_2_ labeling and three for reference (unlabeled or reference plots). The distance between two adjacent plots was at least 2 m. For each labeled plot, we built a 50 cm × 50 cm × 40 cm (length × width × height) chamber composed of a frame covered with polyethylene foil (light transmittance rate: 90%)^19^. To avoid gas leakage, the chamber was sealed by burying part of the foil into soil and sealing with water.

^13^CO_2_ pulse was produced by injecting 10 ml of 4 M H_2_SO_4_ into a vial containing 1.0 g Na_2_^13^CO_3_ (99% abundance). To facilitate a uniform distribution of ^13^CO_2_, a 5-volt fan was installed inside the chamber and used to mix air thoroughly during labeling. ^13^CO_2_ labeling for all the six chambers was applied almost at the same time (i.e. within 3 min) to ensure that the weather conditions were similar during ^13^CO_2_ assimilation. We removed the chambers 3 h after ^13^CO_2_ labeling.

### Sampling and chemical analysis

In each plot, we sampled shoots, roots and soil, and measured soil CO_2_ efflux 1, 4, 6, 12, 20 and 28 days after ^13^CO_2_ labeling. In each plot, we took two shoot samples and two soil cores (6.4 cm in diameter and 30 cm in depth). Shoots were cut in two small circular areas (6.4 cm in diameter) and combined to make a composite sample. For each soil core, soil and roots were sorted carefully. Soils from the two soil cores were combined into one soil sample. Similarly, roots from the two soil cores were combined into one root sample. Soil samples were air-dried, weighed and ball milled before analysis.

Soil CO_2_ efflux was determined by alkali trapping approach^19^. After cutting the shoots, we installed an opaque chamber (6.4 cm in diameter) on the soil surface. Inside the chamber, we placed a graduated beaker containing 4 M NaOH to trap CO_2_ emission from soil for periods of 3, 2, 6, 8 and 8 days. The amount of NaOH was adjusted to ensure that the neutralization did not exceed one third of the NaOH^19^. To quantify the total CO_2_ efflux from soil, CO_2_ trapped in NaOH was analyzed by titrating NaOH against 0.1 M HCl. For δ^13^C analysis of CO_2_ efflux, 2 M SrCl_2_ was added into NaOH to produce SrCO_3_ precipitation for the ^13^C measurement. After neutralization and drying of SrCO_3,_ the ^13^C signature was determined by an isotope ratio mass spectrometer (Delta Plus, Thermo Fisher Scientific, Bremen, Germany) coupled with an elemental analyser (NC 2500, CE Instruments, Milano, Italy).

Before soil C analysis, soil samples were put in a desiccator that contained 10 M HCL for three days to remove carbonates. Then, the samples were neutralized by adding deionized water and dried. The ^13^C signature and total C content of shoots, roots and soil in both labelled and control plots were determined by the isotope ratio mass spectrometer coupled with the elemental analyser.

### C pool calculation

Aboveground C pool consists of shoot C pool and shoot respiration C pool (C loss due to shoot respiration). Shoot C pool was calculated as shoot biomass (g m^−2^) multiplied by shoot C content (%) during the chase period.

Belowground C pool consists of root C pool, soil C pool and soil respiration efflux (C loss due to soil respiration). Root C pool was calculated as root biomass (g m^−2^) multiplied by root C content (%) during the chase period.

Soil C pool (C_soil pool_) was calculated as (1): 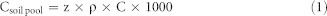
Where z (cm) is thickness of the soil layer, ρ (g cm^−2^) is soil bulk density, and C (%) is soil organic C content^15^,^19^.

Soil respiration C efflux (C_soil res_) was calculated as: 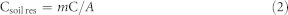
Where *m*C represents the amount of C absorbed in NaOH within the given chase period, and *A* is the surface area covered by the chamber.

### Stable isotope calculation

We calculated the increment of ^13^C at a specific sampling time (*t*) as follows^19^:
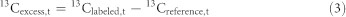
Where ^13^C_excess, t_ is the increment of the ^13^C atom% value at time t, ^13^C_labeled, t_ is the atom% value of a sample in the labeled plot at time t, and ^13^C_reference, t_ is the average atom% value of the samples in the three reference plots at time t.

We also determined ^13^C_i,t_ (g m^−2^), i.e., the amount of ^13^C incorporated into the C-pool i at time t, using the following formula^19^:
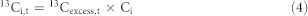
Where C_i_ is the C pool or efflux i (i = shoot, root, soil or soil respiration), which is supposed to be stable (i = shoot, root, soil) or changeable (i = soil respiration) during the whole chase period. C_i_ (i = shoot, root, soil) was calculated by means of biomass and C content during the chase period. Except two sampling periods of the control site, the biomass did not significantly differ between two intervals in the control and waterlogged sites during the chase period (Supplementary FigureS1 online).

The sum of the percentage of recovered ^13^C in shoots and in belowground C pool (including ^13^CO_2_ efflux from soil) represents the recovery of ^13^C in all considered pools at a specific sampling time. At each sampling time, shoot respiration is the missing quantity of ^13^C to 100% ^13^C recovery. Losses by leaching of dissolved organic matter were assumed to be negligible.

Then, we could calculate the ^13^C recovery (A ^13^C_i,t_) in C pool or efflux i (i is shoot, root, soil or soil respiration) at time t, which was referred to the reference recovery of day 1 ( ^13^C_reference, 1_)^19^. The calculation considered the allocation of C fixed in plant tissues and soil at day 1 as described by Hafner et al. 2012. C_reference_ may be underestimated because ^13^C_reference, 1_ was not the real original ^13^C_reference _due to shoot respiration was missing.
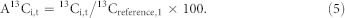
Shoot respiration (A ^13^C_shoot res, t_, % of recovered ^13^C) was estimated by the following equation^19^: 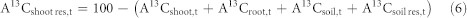


### Statistical analysis

We used ANOVA with repeated measures to examine effects of waterlogging (waterlogging *vs*. control), pool type (shoot, shoot respiration, root, soil and soil respiration) and measuring time on ^13^C partition. In this model, both pool type and measuring time were treated as repeated variables. Pool type was a repeated variable because ^13^C partitions to the five pools in each plot were not independent. We also conducted ANOVA to test effects of waterlogging and measuring time on ^13^C partition in each pool type. All analyses were conducted with SPSS (SPSS, Chicago, IL, USA). Statistically significant difference was set as *p* <0.05 unless stated otherwise.

## Author Contributions

J.Q.G., X.L.X. and F.H.Y. scoped and designed the experiments; J.Q.G., J.J.G., X.W.Z. and Z.H.D. performed the experiments; J.Q.G., X.L.X. and F.H.Y. analyzed the data and wrote the manuscript.

## Supplementary Material

Supplementary Information Supplementary Figures

## Figures and Tables

**Table 1:** Aboveground and belowground stable C stocks and soil respiration in the control and the waterlogged plots

Stock type	Control	Waterlogged	*t*	*P*
Shoot (Mg C ha^−1^)	1.39 ± 0.13	2.41 ± 0.19	5.2	**<0.001**
Root (Mg C ha^−1^)	0.74 ± 0.11	1.68 ± 0.36	2.7	**0.041**
Soil (Mg C ha^−1^)	46.28 ± 4.06	54.37 ± 1.64	1.9	0.203
Soil respiration (Mg C ha^−1^ d^−1^)	0.025 ± 0.003	0.016 ± 0.003	5.6	**0.011**

**Table 2:** Effects of waterlogged, pool type and sampling date on ^13^C partitioning, by repeated measures ANOVA with date as the repeated factor

Effect	*df*	*F*	*P*
(A) For all five C pools			
Waterlogging (W)	1, 4	1.0	0.374
Pool type (P)	4, 64	222.0	**<0.001**
Date (D)	4, 64	1.0	0.436
W × P	4, 64	3.6	**0.029**
W × D	4, 64	1.0	0.436
P × D	16, 64	10.5	**<0.001**
W × P × D	16, 64	2.7	**0.002**
(B) For aboveground *vs.* belowground C pools			
Waterlogging (W)	1, 4	1.0	0.374
Pool type (P)	1, 16	471.9	**<0.001**
Date (D)	4, 16	1.5	0.256
W × P	1, 16	0.7	0.449
W × D	4, 16	1.2	0.350
P × D	4, 16	1.5	0.256
W × P × D	4, 16	1.2	0.350

**Table 3:** Effects of waterlogging and measuring date on ^13^C partition to each of the five C pools

	Waterlogging		Date		Interaction	
	*F*_1,4_	*P*	*F*_4,16_	*P*	*F*_4,16_	*P*
Partition to shoot mass	4.70	0.096	22.88	**<0.001**	3.14	**0.044**
Partition to shoot respiration	1.63	0.271	11.12	**<0.001**	3.35	**0.036**
Partition to root mass	24.56	**0.008**	7.08	**0.002**	4.33	**0.015**
Partition to soil	3.60	0.130	1.44	0.266	1.59	0.224
Partition to soil respiration	2.58	0.183	35.29	**<0.001**	3.66	**0.027**
Partition to aboveground	0.6	0.460	1.5	0.227	1.3	0.321
Partition to belowground	0.6	0.460	1.5	0.227	1.3	0.321

For partition to aboveground and belowground C pools, degree of freedom is (1, 20) for the waterlogging effect, and (4, 20) for the date and interaction effects.
