# Supplementary material for: Effects of waterlogging on carbon assimilate partitioning in the Zoigê alpine wetlands revealed by 13CO2 pulse labeling
Source: Sci Rep. 2015 Mar 23;5:9411. doi: 10.1038/srep09411 (PMC4369740; doi:10.1038/srep09411)

Title: Effects of waterlogging on carbon assimilate partitioning in the Zoigê alpine wetlands revealed by $^{13}CO_2$ pulse labeling

Author list: Jun-Qin Gao[a,*], Ju-Juan Gao[a], Xue-Wen Zhang[a], Xing-Liang Xu[b], Zhao-Heng Deng[a], Fei-Hai Yu[a]

[a] School of Nature Conservation, Beijing Forestry University, Beijing 100083, China

[b] Key Laboratory of Ecosystem Network Observation and Modelling, Institute of Geographic Sciences and Natural Resources Research, Chinese Academy of Sciences, Beijing 100101, China

* Corresponding author

School of Nature Conservation, Beijing Forestry University, Beijing 100083, China

E-mail address: gaojq@bjfu.edu.cn

Phone: +86-10-62336293

Fax: +86-10-62336724

Supplementary Figures

Figure S1 Biomass during the chase period in the control and the waterlogged sites.

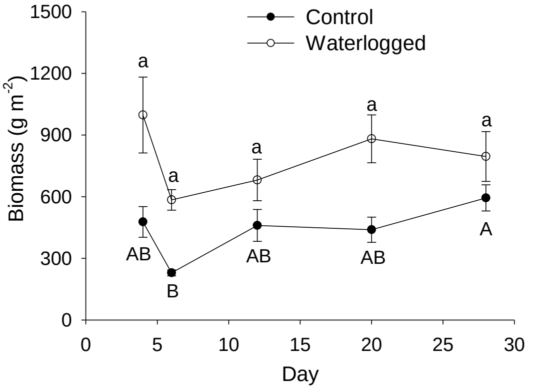

Figure S2 Dynamics of soil moisture content during the chase period.

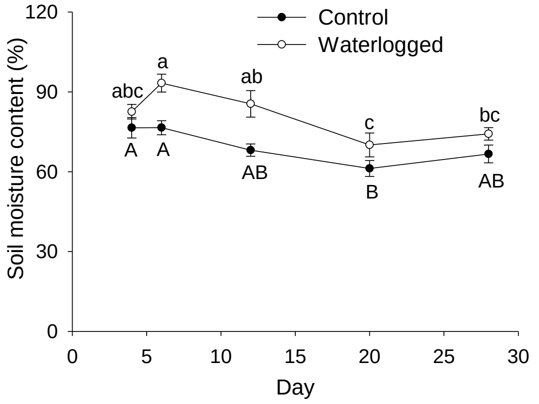

Supplement: Supplementary Information — Supplementary Figures [file srep09411-s1.pdf]
